# Immunological Outcomes of Bovine Colostrum Supplementation in Trained and Physically Active People: A Systematic Review and Meta-Analysis

**DOI:** 10.3390/nu12041023

**Published:** 2020-04-08

**Authors:** Natalia Główka, Krzysztof Durkalec-Michalski, Małgorzata Woźniewicz

**Affiliations:** 1 Institute of Human Nutrition and Dietetics, Poznań University of Life Sciences, Wojska Polskiego 31, 60-624 Poznań, Poland; durkmich@up.poznan.pl (K.D.-M.); malgorzata.wozniewicz@up.poznan.pl (M.W.); 2 Department of Food and Nutrition, Poznan University of Physical Education, Królowej Jadwigi 27/39, 61-871 Poznań, Poland

**Keywords:** colostrum, immunology, physical activity, sport, immunity support

## Abstract

Bovine colostrum (BC) is a promising natural product applied to improve immunological functions. However, there is very little evidence on the true benefits of BC treatment on the immune function of trained and physically active people; moreover, there is no consensus on the supplementation strategy. For this reason, the aim of this meta-analysis was to quantify the effects of BC supplementation on immunological outcomes in physically active people. Data from 10 randomised controlled trials (RCTs) investigating the effect of BC supplementation in athletes and physically active adults were analysed, involving 239 participants. The results show that BC supplementation has no or a fairly low impact on improving the concentration of serum immunoglobulins (IgA, IgG), lymphocytes and neutrophils, and saliva immunoglobulin (IgA) in athletes and physically active participants. Previous research has shown BC to reduce upper respiratory tract infections; nevertheless, there is a gap of scientific knowledge on the mechanisms underlying these effects. Future RCTs are needed to focus on finding these mechanisms, as well as on preparing a clear consensus on a BC supplementation strategy in trained athletes and the physically active population.

## 1. Introduction

Moderate physical activity and recreational sports training may improve immunological functions and reduce the risk of infections [1,2,3,4]. Nevertheless, strong evidence-based research confirms that athletes involved in intensive or prolonged physical training are more susceptible to infections, especially upper respiratory tract symptoms. A higher incidence of infections is often observed in endurance athletes (mainly triathletes, swimmers, cyclists) [1]. Moreover, problems with immune disturbances may compromise training and competition performance [1,2,3,4]. Hence, it is important to elaborate different strategies to improve the immunological functions, like nutrition or supplementation.

The crucial role of the immune system is to protect the human body from pathogens and issues with infections. In this way, it is responsible for maintaining homeostasis. In this respect, both the non-specific innate system and the specific adaptive immune system cooperate in order to determine a state of immunity against infection [1]. It has been observed that some components of the immune system are suppressed after exercise, which can last from a few hours to even a few days [2,4]. Exercise-induced immunodepression may occur if subsequent exercise is commenced before full recovery of immune efficacy. Due to the large volume of intense effort that endurance athletes undergo (especially swimmers and triathletes), they are at a high risk of developing an immunological disorder, especially upper respiratory tract infections (URTI) such as the common cold [4]. A possible mechanism explaining increased susceptibility to infections is termed ‘open window’ [1]. Nevertheless, changes in isolated immune markers may not always be observed or used to predict the risk of illnesses [1]. Biomarkers that are used by nutritional immunologists in the field of exercise immunology are divided into five categories (upper respiratory tract illness, in vivo immunity, mucosal immunity, ex vivo/in vitro immunity, immune cell trafficking and other markers) based on different methods [1,4,5].

It is important to underline that, among the various supplements, bovine colostrum (BC) seems to induce beneficial effects via the improvement of immune function. BC is a substance produced naturally by the mammary glands of mammals for 24–72 h after calving. The significant impact of BC intake on the development of the immune system of calves has led to the use of BC-based products in humans [5,6,7,8]. Evidence suggests that BC may have many clinical or therapeutic applications in humans [9]. There are limited studies concerning the supplementation of BC in athletes in order to positively affect the immune system. Currently, there is only one systematic review and meta-analysis of five randomised controlled trials (RCTs) showing that oral supplementation of BC can reduce the incidence rate of URTI days and episodes in athletes [10]. In terms of immunological biomarkers, studies are inconclusive. In one of them, a 33% increase in SIgA concentration in saliva was observed after two weeks of 20 g BC supplementation [11]. In the latter study, the use of a chocolate drink containing 10 g BC in a group of recreational runners for a 12-week period led to a 79% increase in resting SIgA concentrations [12]. On the contrary, some studies have found no significant difference in saliva SIgA concentrations between supplemented and placebo (PLA) groups [13,14,15,16,17,18,19]. However, more recent studies have found beneficial effects of BC treatment by stimulating the neutrophil oxidative burst, blunting the prolonged exercise-induced decrease in in vivo immune responsiveness to a novel antigen and reducing exercise-induced muscle damage and markers of inflammation [20,21].

Although BC could indirectly improve athletic performance by improving immunity, to the best of the authors’ knowledge, there is no clear consensus on the supplementation strategy. There is also very little evidence of the true benefits of BC supplementation with regard to immune function. Therefore, we carried out a systematic review and final meta-analysis of relevant articles published in the literature to test the hypothesis that the effects of BC on athletes’ immunological outcomes outperform placebo (PLA) and there would be an association between the dose and time of supplementation and the effect size. In order to conduct the meta-analysis, we focused on the most accessible and frequently used immunological markers evaluated in studies on trained and physically active people.

## 2. Materials and Methods 

### 2.1. Searching Strategies

The present article is a systematic review with a meta-analysis focusing on the effect of BC on the immunity of athletes. The systematic review protocol was registered in the PROSPERO—International Prospective Register of Systematic Reviews with the registration number CRD42019125404 [22]. A search of the literature was conducted by electronic search for original papers of four literature databases (PubMed, Web of Science, Scopus and SPORTDiscus). The search included original papers written in English and published before 14th February 2019; no year restriction was applied to the search strategy. The extraction was restricted to randomised controlled trials. The following index terms were used: ((‘colostrum’ [All Fields]) AND (‘immune’ [All Fields] OR ‘immunity’ [All Fields] OR ‘IgA’ [All Fields] OR ‘immunoglobulins’ [All Fields] OR ‘growth factors’ [All Fields] OR ‘IGF-1’ [All Fields] OR ‘interleukins’ [All Fields]) AND (‘exercise’ [All Fields] OR ‘performance’ [All Fields] OR ‘training’ [All Fields] OR ‘active’ [All Fields] OR ‘trained’ [All Fields] OR ‘healthy’ [All Fields])).

### 2.2. Inclusion and Exclusion Criteria

For the articles obtained in the database search, the following inclusion criteria were applied to select the final studies: intervention studies (randomised double-blind placebo-controlled trials (DB-RCTs), English-language articles and studies conducted in adult, male and female, trained and/or physically active subjects that were being supplemented with BC. Moreover, we also analysed papers that had taken into consideration relevant and the most commonly reported immunological markers in BC studies on trained and physically active people.

The exclusion criteria comprised: studies performed in specific, diseased group of patients, pregnant or breast-feeding women, infants, adults not involved in regular physical activity or in animals; studies carried out using non-bovine colostrum (e.g., human colostrum), hyper-immune colostrum, mixed colostrum product or only one colostrum ingredient (e.g., lactoferrin), not whey PLA; articles available only in abstract form (not possible to contact the authors).

### 2.3. Data Extraction

The data in the studies derived from the databases were evaluated by one investigator (N.G.) using a predefined data sheet. The extraction was checked independently by two other authors (M.W., K.D.-M.). First, a list of all potential papers was downloaded; second, all duplicates were deleted; third, titles and abstracts were screened to identify studies that potentially met the eligibility criteria; fourth, full texts were subsequently assessed for eligibility. Subsequently, disagreements were resolved through discussion until a consensus was achieved. Each selected publication was studied critically. If the publications included for full-text analysis were not available in the full version, their authors were contacted directly.

### 2.4. Quality Assessment of the Experiments

**Figure 1:**
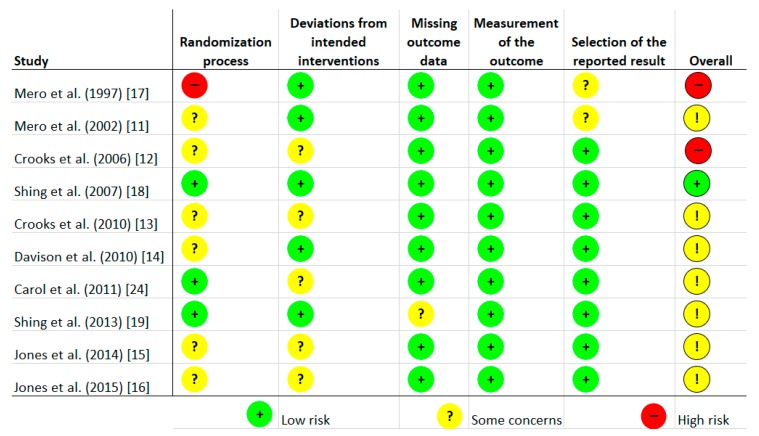
Risk of bias of included studies.

**Figure 2:**
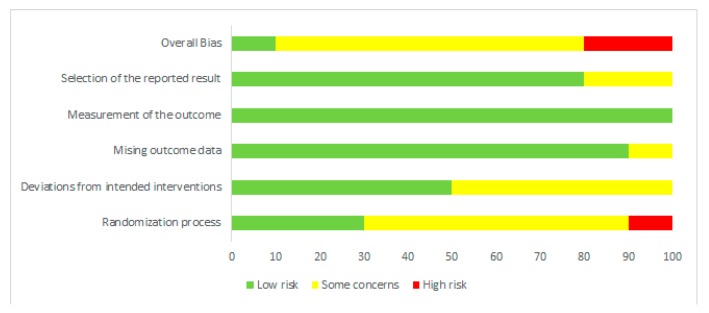
Risk of bias summary: review authors’ judgements about each risk of bias item for each included study.

Risk of bias was assessed independently by two investigators using the latest version of the Cochrane Collaboration Risk-of-Bias tool (RoB, 2 March 2019) [23] in randomised trials. Studies were assessed in five domains: bias arising from the randomisation process; bias due to deviations from intended interventions; bias due to missing outcome data; bias in measurement of the outcome; bias in selection of the reported result. The tool includes algorithms that map responses to signalling questions onto a proposed risk-of-bias judgement for each domain in three levels: low risk of bias, some concerns, and high risk of bias. The highest concerns were found in relation to randomisation process, where random sequence generation was characterised as low risk only in three studies [18,19,24]. The bias in the remaining studies were unclear [11,12,13,14,15,16] or high [17]. To sum up, overall, seven studies were characterised as unclear risk of bias [11,13,14,15,16,19,24], two trials as high risk [12,17], and one study as low risk [18]. Full details are given in Figure 1 and Figure 2.

### 2.5. Statistical Analysis

The data obtained were expressed as the mean ± standard deviation (SD). A meta-analysis was performed throughout to synthesise the data from DB-RCTs. Not all studies provided adequate data for inclusion and analysis; therefore, authors were contacted via e-mail. In other cases, data were converted according to method proposed by Hozo et al. [25]. The data were analysed using a random-effects model, which allowed that the true effect could vary from study to study. The effect sizes in the studies were assumed to represent a random sample of these effect sizes. The effect size was investigated using Hedges’ *g* (corrected due to small sample sizes) with a 95% confidence interval (CI). The analyses were performed using statistical software (Statistica 13.3, Software Inc., Cracow, Poland). To examine potential sources of heterogeneity, subgroup analysis of several potential moderator variables (supplemented dose, and time of supplementation) were undertaken. The results of the meta-analysis were visualised using a forest plot which illustrates the results of the individual studies and the summary effect.

Funnel plots were constructed to estimate the effect of a publication bias. The funnel plot reveals the relationship between the effect size *g* of each trial and its corresponding standard error of the mean difference. *p* Value < 0.05 was considered as significant. Data are presented in (Supplementary Resource 1 Figures S1–S8). 

## 3. Results

### 3.1. Main Search

**Figure 3:**
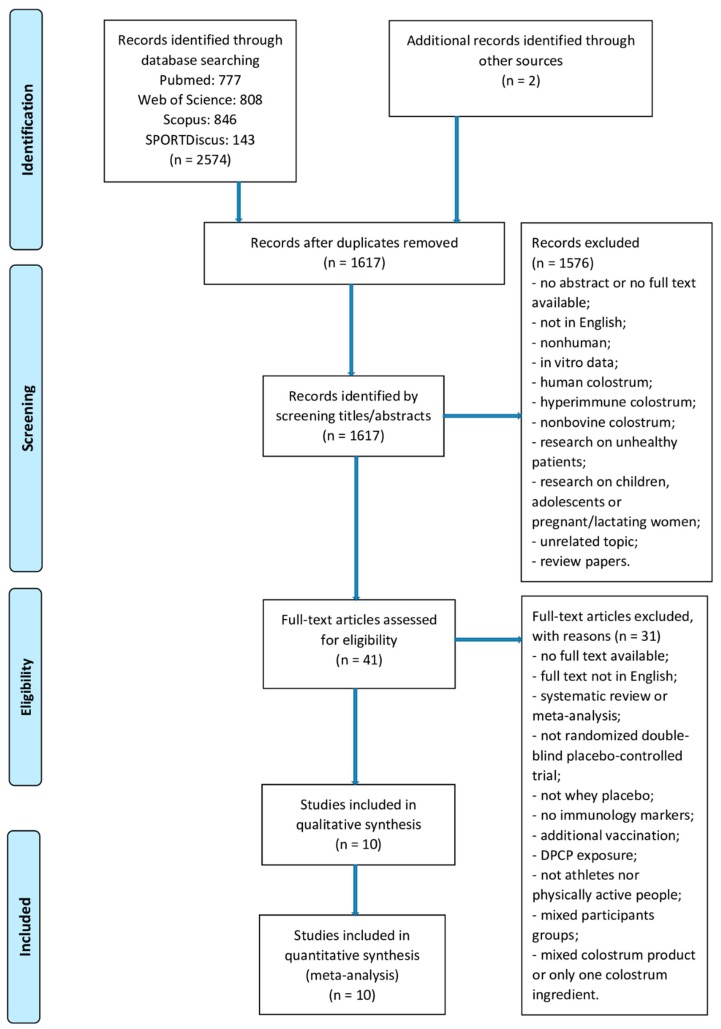
Study selection process.

The search identified 2576 potential trials (PubMed: *n* = 777, Web of Science: *n* = 808, Scopus: *n* = 846, SPORTDiscus: *n* = 143). Two additional articles were identified through other sources. After removal of 959 duplicates, 1617 records underwent title and abstract screening, 41 articles underwent full-text screening. Finally, only 10 RCTs met the inclusion criteria and were included in the final analysis [11,12,13,14,15,16,17,18,19,24]. The study selection process is shown in Figure 3.

### 3.2. Study Characteristics

Ten RCTs with 239 patients were included (82% males). Their characteristics are presented in Table 1 The studies were published between 1997 and 2015. Of the ten studies, three were conducted in the UK [14,15,16], two in Australia [18,19], two in New Zealand [12,13], two in Finland [11,17], and one in the Netherlands [24]. One of these trials was explicitly identified in the study report as being a pilot study [19]. One of the trials used two different BC doses [17]. Sample sizes ranged from nine to 53. Participants within six trials were athletes regularly involved in training [11,12,13,17,18,19,24], whether within three trials participants were recreationally active [14,15,16]. Participants were trained sprinters, jumpers, track and field athletes, country skiers, orienteers, cyclists, runners, or swimmers. Aside from the supplementation, participants in all of the trials continued with their usual diets during the trial period. Common participant exclusion criteria in the individual trials were the use of supplements [11,12,13,15,16,17,18], lactose intolerance [12,13], use of medication [12,13,15,16,24], or dairy allergy [12,13,16,24]. Two studies [15,16] also stipulated that participants should be non-smokers and two studies [15,24] stipulated that participants should be free from any infectious illness for ≥4 weeks. All trials compared BC to PLA. The supplementation periods of these trials ranged from 8 days to 12 weeks. Nine studies [11,12,13,14,15,16,18,19,24] included provision of BC in powdered form and one [17] in liquid form; the daily dosage of the BC intervention ranged from 10 to 25 g in powder and 25/125 ml in liquid per day. In two trials, the PLA comprised of whey protein [18,19], three trials used skimmed milk [12,13,24], one trial used maltodextrins [11], one trial used normal milk [17], and three trials used a product isoenergetic and isomacronutrient to BC [14,15,16], consumed at the identical dose, frequency, and duration as the BC supplement. Although each study reported to be a double-blind trial, none assessed the extent to which participants may have deduced their group allocation/treatment regimen.

### 3.3. Effect of Bovine Colostrum (BC) on Immunological Outcomes

#### 3.3.1. Lymphocytes Pre-Exercise

**Figure 4:**
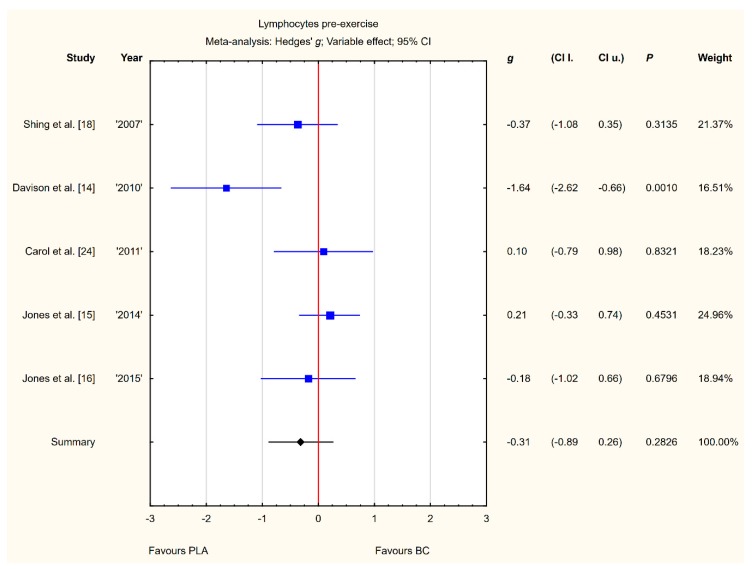
Forest plot of the random-effects meta-analysis of changes in lymphocytes concentration. Heterogeneity: Tau^2^ = 0.27; Chi^2^ = 11.13; df = 4; I^2^ = 64.06%; *p* = 0.0252. Abbreviations: BC–bovine colostrum, PLA–placebo.

Five trials reported pre-exercise lymphocyte concentrations in the blood after BC supplementation [14,15,16,18,24]. Within these studies, 139 participants were included in the analysis (68 participants in the BC group and 71 in the PLA group). The point estimate of effect only for two of the included trials indicated a higher lymphocyte concentration with BC. Pooled analyses from the five trials did not demonstrate a significant effect of BC supplementation on the lymphocyte concentration (*g* Hedges −0.31, 95% confidence −0.89 to 0.26, *p* value 0.2826). A substantial level of statistical heterogeneity was detected among trial level effects when all five trials were included (Figure 4). 

#### 3.3.2. Neutrophils Pre-Exercise

**Figure 5:**
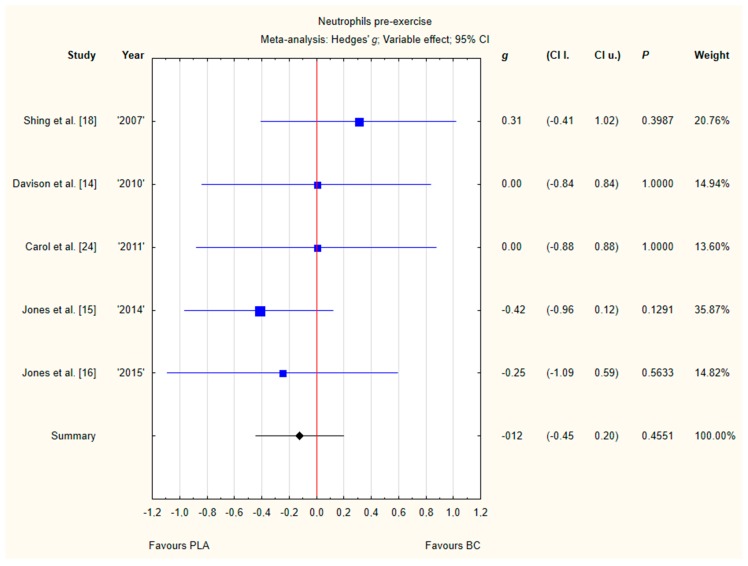
Forest plot of the random-effects meta-analysis of changes in neutrophils concentration. Heterogeneity: Tau^2^ = 0; Chi^2^ = 2.79; df = 4; I^2^ = 0%; *p* = 0.5933. Abbreviations: BC–bovine colostrum, PLA–placebo.

Five trials reported pre-exercise neutrophil concentrations in the blood after BC supplementation [14,15,16,18,24]. Within these studies, 139 participants were included in the analysis (68 participants in the BC group and 71 in the PLA group). The point estimate of effect for only one of the included trials indicated a higher neutrophil concentration with BC. Pooled analyses from the five trials did not demonstrate a significant effect of BC supplementation on the neutrophil concentration (*g* Hedges −0.12, 95% confidence −0.45 to 0.20, *p* value 0.4551). No important heterogeneity was detected among trial level effects when all five trials were included (Figure 5). 

#### 3.3.3. Serum IgA Pre-Exercise

**Figure 6:**
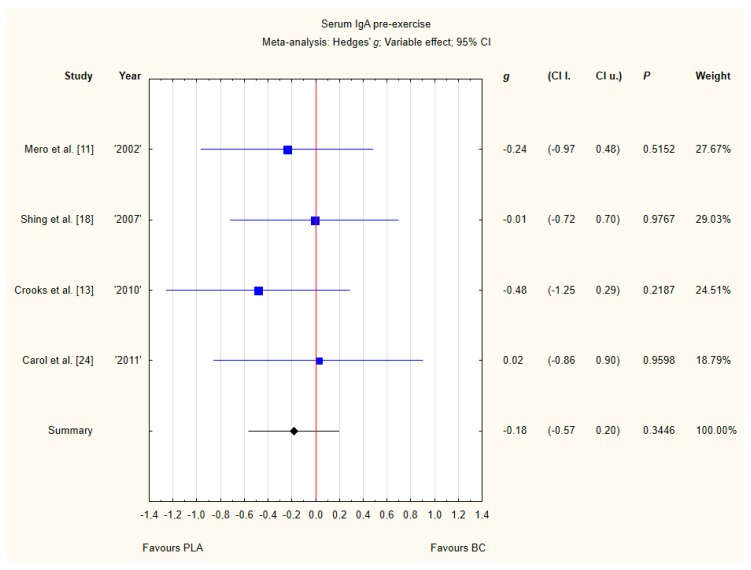
Forest plot of the random-effects meta-analysis of changes in IgA concentration. Heterogeneity: Tau^2^ = 0; Chi^2^ = 1.05; df = 3; I^2^ = 0%; *p* = 0.79. Abbreviations: BC–bovine colostrum, IgA–Immunoglobulin A, PLA–placebo.

Four trials reported pre-exercise IgA concentrations in the blood after BC supplementation [11,13,18,24]. Within these studies, 102 participants were included in the analysis (54 participants in the BC group and 48 in the PLA group). The point estimate of effect for none of the included trials indicated a higher IgA concentration with BC. Pooled analyses from the five trials did not demonstrate a significant effect of BC supplementation on the IgA concentration (*g* Hedges −0.18, 95% confidence −0.57 to 0.20, *p* value 0.3446). No important heterogeneity was detected among trial level effects when all four trials were included (Figure 6).

#### 3.3.4. Serum IgG Pre-Exercise

**Figure 7:**
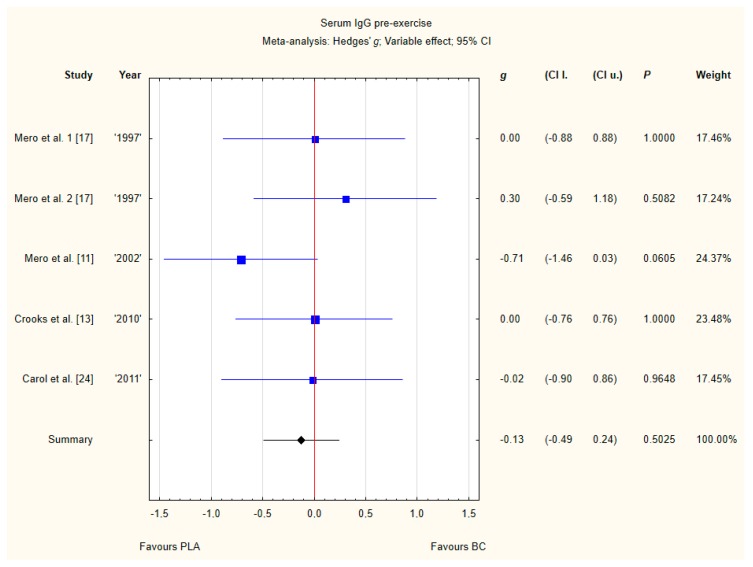
Forest plot of the random-effects meta-analysis of changes in IgG concentration. Heterogeneity: Tau^2^ = 0; Chi^2^ = 3.51; df = 4; I^2^ = 0%; *p* = 0.4756. 1–the first dose from Mero et al. [17] 1997 study, 2–the second dose from Mero et al. 1997 [17] study. Abbreviations: BC–bovine colostrum, IgG–Immunoglobulin G, PLA–placebo.

Four trials reported pre-exercise IgG concentrations in blood after BC supplementation [11,13,17,24]. Within these studies 102 participants were included in the analysis (54 participants in the BC group and 48 in the PLA group). The point estimate of effect only for one of the included trials indicated a higher IgG concentration with BC. Pooled analyses from the five trials did not demonstrate a significant effect of BC supplementation on the IgG concentration (*g* Hedges −0.18, 95% confidence −0.57 to 0.20, *p* value 0.3446). No important heterogeneity was detected among trial level effects when all four trials were included (Figure 7).

#### 3.3.5. SIgA Pre-Exercise

**Figure 8:**
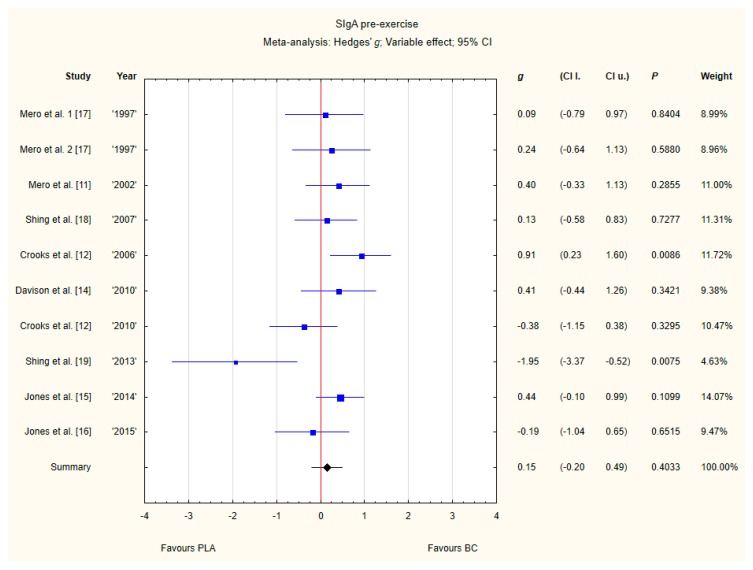
Forest plot of the random-effects meta-analysis of changes in pre-exercise SIgA concentration. Heterogeneity: Tau^2^ = 0.15; Chi^2^ = 17.35; df = 9; I^2^ = 48.13%; *p* = 0.0435. 1–the first dose from Mero et al. [17] 1997 study, 2–the second dose from Mero et al. [17] 1997 study. Abbreviations: BC–bovine colostrum, SIgA–secretory Immunoglobulin A, PLA–placebo.

Nine trials reported pre-exercise SIgA concentrations in saliva after BC supplementation [11,12,13,14,15,16,17,18,19]. Within these studies, 257 participants were included in the analysis (130 participants in BC group and 127 in the PLA group). The point estimate of effect for seven of the included trials indicated a higher SIgA concentration with BC. Nevertheless, pooled analyses from the nine trials demonstrated a non-significant effect of BC supplementation on the SIgA concentration (*g* Hedges 0.15, 95% confidence −0.20 to 0.49, *p* value 0.4033). A moderate level of statistical heterogeneity was detected among trial level effects when all nine trials were included (Figure 8).

#### 3.3.6. SIgA Post-Exercise

**Figure 9:**
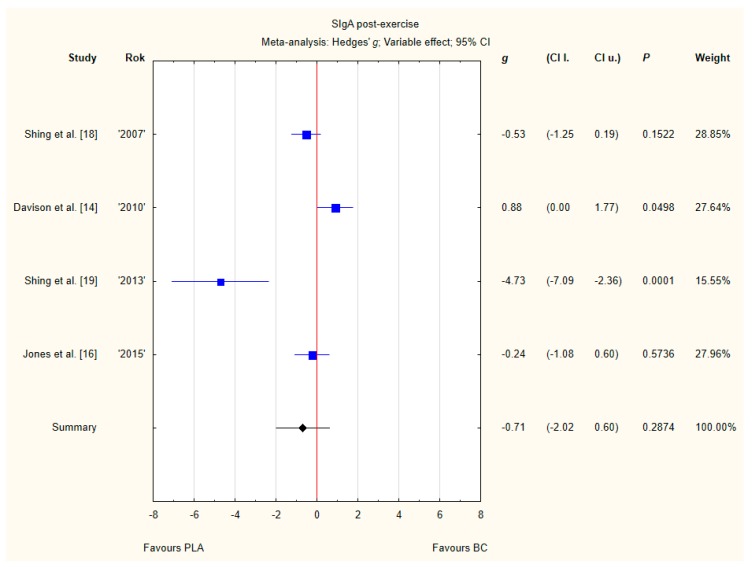
Forest plot of the random-effects meta-analysis of changes in post-exercise SIgA concentration. Heterogeneity: Tau^2^ = 1.4; Chi^2^ = 20.64; df = 3; I^2^ = 85.47%; *p* = 0.0001. Abbreviations: BC–bovine colostrum, SIgA–secretory Immunoglobulin A, PLA–placebo.

Four trials reported post-exercise SIgA concentration in saliva after BC supplementation [14,16,18,19]. Within these studies 79 participants were included in the analysis (38 participants in the BC group and 41 in the PLA group). The point estimate of effect only for one of the included trials indicated a higher SIgA concentration with BC. Pooled analyses from the four trials did not demonstrate a significant effect of BC supplementation on the SIgA concentration (*g* Hedges −0.71, 95% confidence −2.02 to 0.60, *p* value 0.2874). A substantial level of statistical heterogeneity was detected among trial level effects when all four trials were included (Figure 9).

#### 3.3.7. Pre-Exercise Siga Concentration Changes from Baseline to Post-Supplementation

**Figure 10:**
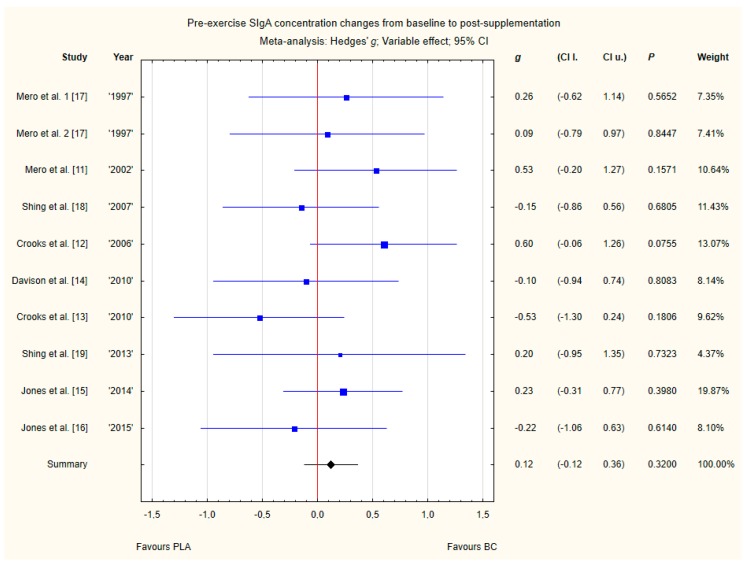
Forest plot of the random-effects meta-analysis of changes from baseline to post-supplementation in pre-exercise SIgA concentration. Heterogeneity: Tau^2^ = 0; Chi^2^ = 7.65; df = 9; I^2^ = 0%; *p* = 0.57. 1–the first dose from Mero et al. [17] 1997 study, 2–the second dose from Mero et al. [17] 1997 study. Abbreviations: BC–bovine colostrum, SIgA–secretory Immunoglobulin A, PLA–placebo.

Nine trials reported pre-exercise saliva SIgA concentration changes from baseline to post-supplementation [11,12,13,14,15,16,17,18,19]. Within these studies, 257 participants were included in the analysis (130 participants in the BC group and 127 in the PLA group). The point estimate of effect for six of the included trials indicated positive changes with BC (a higher SIgA concentration). Nevertheless, pooled analyses from the nine trials demonstrated a non-significant effect of BC supplementation on the SIgA concentration changes (*g* Hedges 0.12, 95% confidence −0.12 to 0.36, *p* value 0.3200). No important heterogeneity was detected among trial level effects when all nine trials were included (Figure 10).

#### 3.3.8. Post-Exercise SIgA Concentration Changes from Baseline to Post-Supplementation

**Figure 11:**
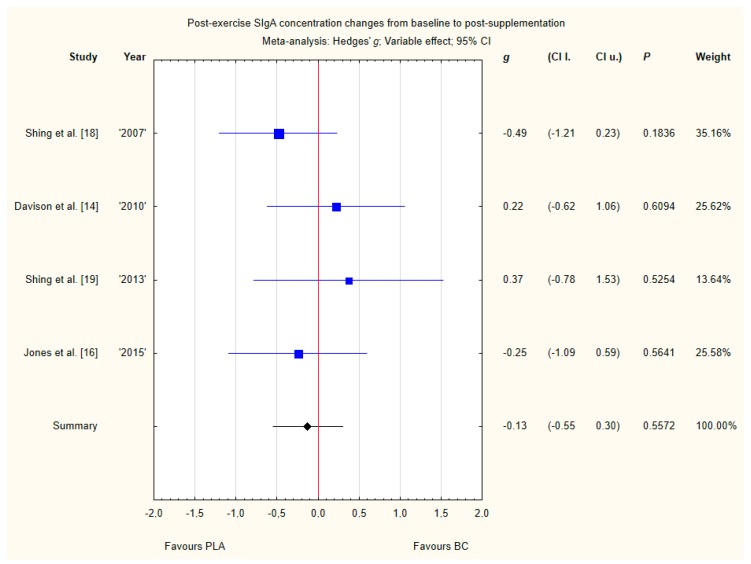
Forest plot of the random-effects meta-analysis of changes from baseline to post-supplementation in post-exercise SIgA concentration. Heterogeneity: Tau^2^ = 0; Chi^2^ = 2.42; df = 3; I^2^ = 0%; *p* = 0.4898. Abbreviations: BC–bovine colostrum, SIgA–secretory Immunoglobulin A, PLA–placebo.

Four trials reported post-exercise saliva SIgA concentration changes from baseline to post-supplementation [14,16,18,19]. Within these studies, 79 participants were included in the analysis (38 participants in the BC group and 41 in the PLA group). The point estimate of effect for two of the included trials indicated increase in SIgA concentrations with BC. Pooled analyses from the four trials did not demonstrate a significant effect of BC supplementation on SIgA concentration changes (*g* Hedges −0.13, 95% confidence −0.55 to 0.30, *p* value 0.5572) (Figure 11). No important heterogeneity was detected among trial level effects when all four trials were included.

### 3.4. Subgroup Analyses

**Figure 12:**
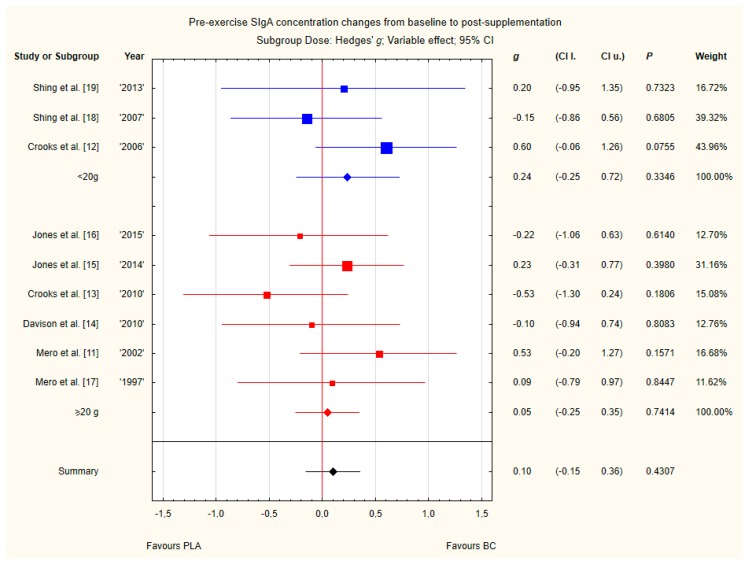
Forest plot of the random-effects meta-analysis of changes from baseline to post-supplementation in pre-exercise SIgA concentration in subgroups regarding BC dose. Test for subgroup differences: Chi^2^ = 0.42; df = 1; *p* = 0.52. Abbreviations: BC–bovine colostrum, SIgA–secretory Immunoglobulin A, PLA–placebo.

**Figure 13:**
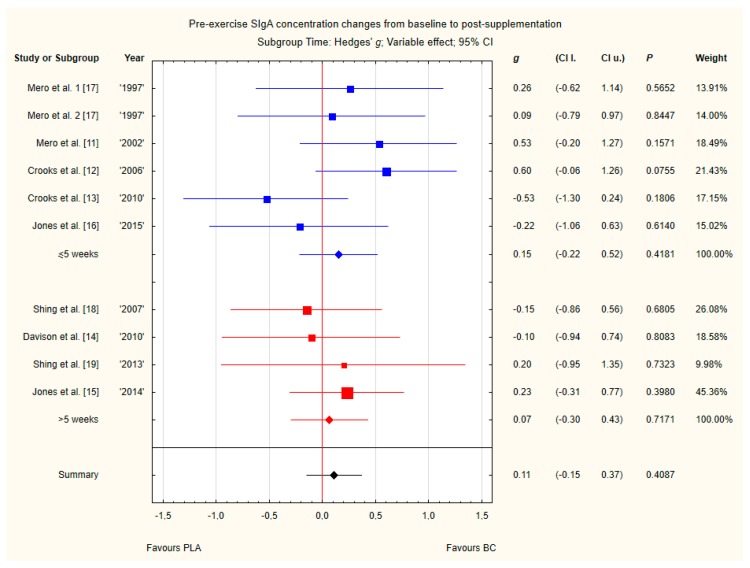
Forest plot of the random-effects meta-analysis of changes from baseline to post-supplementation in pre-exercise SIgA concentration in subgroups regarding BC supplementation time. Test for subgroup differences: Chi^2^ = 0.1; df = 1; *p* = 0.75. 1–the first dose from Mero et al. [17] 1997 study, 2–the second dose from Mero et al. [17] 1997 study. Abbreviations: BC–bovine colostrum, SIgA–secretory Immunoglobulin A, PLA–placebo.

Subgroup analyses taking into account the supplemented BC dose and duration of supplementation did not show any significant differences (Figure 12 and Figure 13). However, there was a tendency, but not statistically significant, for higher SIgA concentrations in groups with doses above 20 g of BC.

### 3.5. Sensitivity Analyses

I^2^ was used to evaluate between-study heterogeneity. Values of *I*^2^, interpreted on the basis of general methods for Cochrane reviews [26], reflected moderate, substantial, and considerable heterogeneity. 

## 4. Discussion

This review identified ten randomised controlled trials in trained and physically active people [11,12,13,14,15,16,17,18,19,24], evaluating the effects of BC on selected immunological markers. The findings of this meta-analysis did not show any statistically significant impact of BC supplementation on the concentrations of selected immunoglobulins (IgA, IgG) in blood, secretory immunoglobulin A (SIgA) in saliva, or selected leukocytes in blood (lymphocytes, neutrophils).

There is currently no meta-analysis that has assessed the effect of BC on immunological outcomes in trained and physically active people. We are not aware of another systematic review evaluating the effect of BC on immunity in athletes, although one review discussed the clinical applications of BC therapy [9] and one review discussed the effect of BC on upper respiratory symptoms (URS) in healthy active adults [10]. In a recently published meta-analysis by Jones et al. [10], it was shown that supplementation with BC has a significant effect in reducing the rate of URS days and episode rates of URS, compared to placebo. Nevertheless, in our analysis, we focused on the most commonly reported immunological markers in BC studies on trained and physically active people to consider their significance in explaining the effects observed in these previous studies. Whereas the supplementation dosage was similar among studies (10–20 g/day of BC), diversity in the supplementation strategy (duration, dose, spreading the dose), as well as sample size, time of blood and saliva collection, renders the comparison between interventions difficult. Finally, we identified an important gap in the literature relating to the mechanisms responsible for the impact of BC on immunity, as well as the limited number of good quality studies examining BC supplementation in trained and physically active people. 

The selection of markers used to assess immune functions for the most number of studies is mostly done not in the context of health maintenance, but in the context of diseases. Despite this, researchers applied similar criteria to determine the usefulness of these markers. These criteria include clinical relevance, biological sensitivity or practical aspects for use, which is thoroughly described in the work by Albers et al. [5]. Nevertheless, even more specific biomarkers are used in the field of exercise immunonutrition, it is not common in BC studies on athletes. It is dictated by the inevitability of performing the investigations in the field, which forces the use of practical and low-cost measurement tools [4]. Unfortunately, basal markers used in these studies are insensitive and difficult to interpret in specific populations. On the other hand, it should be pointed that more useful markers have not been validated and it is hard to compare them between laboratories [5]. Five categories of biomarkers used in exercise immunonutrition studies based on different methods include: upper respiratory tract illness (e.g., Jackson common cold questionnaire), in vivo immunity (e.g., DTH skin test), mucosal immunity (saliva and tear fluid SIgA), ex vivo/in vitro immunity (e.g., phagocytosis, oxidative burst assays), immune cell trafficking and other markers (e.g., white blood cell count, lymphocyte count, cytokines production) [1,4]. In our meta-analysis we assessed five biomarkers (IgA, IgG, SIgA, lymphocytes, neutrophils) due to the fact that these markers were routinely assessed in studies on the effect of BC on athletes and physically active people. However, we would like to highlight that there are a few studies assessing more clinically relevant biomarkers in BC studies, like neutrophil/lymphocyte surface markers, NK cell cytotoxicity [18], neutrophil oxidative burst [15,16], neutrophil functional capacity, salivary lysozyme release [14], but the amount of them is not enough to include in a meta-analysis.

The impact of exercise concerns aspects of innate, acquired, and mucosal immunity [1]. Innate immunity perturbations are especially seen in the number of circulating leukocytes, which was first observed in reports based on scientific research from over a century ago. Circulating immune cells consist of granulocytes (neutrophils, basophils, eosinophils), lymphocytes (natural killer cells, helper T cells, cytotoxic T cells, B cells) and dendritic cells. Studies have confirmed that leukocytosis (mainly neutrophilia and lymphocytosis) may occur during and immediately post-exercise, which is dependent on the duration and intensity of the exercise (especially with prolonged exercise) [27,28]. After lymphocytosis, during the recovery phase, lymphocytopenia can be observed. An indicator of the overall stress response to exercise may be an increase in the neutrophil:lymphocyte ratio [1]. Moreover, exercise has an impact on the adaptive immune system. The Th1/Th2 balance can be modulated by decreasing the proportion of Th1 cells. This process is proposed as an explanation for increased susceptibility to URS [29,30]. Nevertheless, sparse studies have investigated the effect of BC on the neutrophil:lymphocyte ratio or the Th1/Th2 balance nor post-exercise immunological outcomes in physically active people. Therefore, these results were not included in this meta-analysis (except post-exercise SIgA concentration in saliva). 

Immunoglobulins are crucial for antigen binding and elimination or activation processes [31]. Serum immunoglobulins consist of four main classes (IgA, IgG, IgE, IgM). Based on the literature, it has been shown that a decreased IgG_2_ concentration may be associated with an increased bacterial infection risk. Moreover, there is a strong positive correlation between IgG_2_ and the ability to produce antibodies [32]. Sports medicine studies have revealed a possible decrease in the concentration of IgG_2_ associated with an intensive training period [33] or an intensive bout of exercise [31]. The literature on serum immunoglobulin concentration changes following exercise is conflicting. It is generally believed that there may be some disturbances in immunoglobulin concentrations, especially low levels in elite athletes during the competitive season and significantly impaired levels within hours after competition [34,35].

The common mucosal immune system is considered to be the first line of defence; local production of SIgA is considered to be the major effector of this system [1]. SIgA is also the most investigated parameter in terms of mucosal immunity during exercise. Salivary concentrations are susceptible to the intensity and duration of exercise [36]. It has been observed that saliva SIgA decreases or remains unchanged after prolonged exercise, with the most significant impact following high-intensity and endurance exercise [37,38]. It is believed that athletes suffering from IgA deficiency may contract URS regularly [39]. On the other hand, an increase in SIgA may be the primary mechanism for the decreased URS risk in athletes [40]. In vitro studies have shown that one of the BC components, TGF-β, may stimulate human lymphocytes and promote IgA biosynthesis and secretion [41,42,43]. In animal models, BC protein supplementation has led to elevated secretory immunoglobulins in the gut, which suggests an impact of BC on mucosal immunity [44,45]. Human studies have shown increased SIgA responses to pathogens after BC supplementation [46]. Since BC contains SIgA, studies using BC supplementation measuring SIgA in saliva samples were designed to avoid cross-reactivity against bovine proteins. It was found that the increase in saliva SIgA concentrations in the examined participants was due to the stimulation of unknown mechanisms and was not influenced by the absorption of bovine IgA [12].

### 4.1. Effect of BC on Leukocyte Concentrations

The first study examining the effect of BC on leukocytes [14] did not show any differences between groups in circulating neutrophil and lymphocyte counts. This finding is in line with the results obtained in studies by Shing et al. in 2007, Carol et al. in 2011, and Jones et al. in 2014 and 2015 [15,16,18,24]. It is worth noting that not all studies measured the impact of BC on pre- and post-exercise leukocytes concentration [14,16,18,24]. The results obtained in this meta-analysis concern only pre-exercise levels of leukocytes and suggest that BC supplementation has no benefits in inducing changes in the leukocyte count. 

### 4.2. Effect of BC on Serum Immunoglobulin Concentrations

In the experimental studies of BC supplementation on circulating total serum IgG or IgA concentrations, no significant differences were observed compared to placebo [11,13,17,24]. Nevertheless, it is worth mentioning that in the study by Shing et al. [18], the authors measured different subclasses of IgG and observed the prevention of post-exercise decrease in serum IgG_2_ concentrations. Unfortunately, this result could not be evaluated in this meta-analysis, due to the authors did not mention the total number of IgG. Moreover, the described effects were seen only during acute exercise after a period of prolonged stress, but any effect of BC treatment compared with PLA was recorded after acute exercise during periods of normal training. Furthermore, the results obtained in our meta-analysis suggest that BC supplementation is not effective in inducing changes in serum immunoglobulin concentrations.

### 4.3. Effect of BC on SIgA Concentrations

Studies investigating the impact of BC supplementation on salivary SIgA concentrations showed inconclusive results. The first study on BC impact on SIgA, published by Mero et al. [17] in 1997 did not reveal any significant effect of supplementation, whereas the second study from 2002 [11] showed a 33% SIgA increase in the experimental BC group, but not in the PLA group. Nevertheless, it is important to note that, in this study, the authors used maltodextrin as the placebo, and therefore did not have the same macronutrient profile as the intervention. However, Crooks et al. [12] in 2006 also demonstrated that 10 g/day of BC over a 12-week period can significantly increase SIgA concentrations compared to skim milk PLA. On the other hand, Shing et al. [18] reported no increase in resting SIgA in highly-trained cyclists after 10 g/day of BC for 12 weeks. Similarly, Crooks et al. [13] in 2010 supplemented 20 g/day of BC for 10 weeks and did not notice any significant effects on SIgA. A study by Davison et al. [14] with 20 g/day of BC lasting 4 weeks also did not show any differences between groups in the exercise-induced changes in salivary IgA concentrations. A latter study by Shing et al. [19] did not show any significant differences in SIgA between groups after 8 weeks of 10 g/day of BC vs. PLA, when analysing the values after a period of normal scheduled training. Furthermore, the authors noticed significantly lower values of SIgA in the evening on days 3 and 5 of the race in the BC group. Due to the methodological differences (circadian variations) and the specific race test, values used in this meta-analysis concerned only samples taken on day 1. The lack of a significant effect of BC on salivary SIgA was confirmed in two studies by Jones et al. [15,16] after 4 or 12 weeks of supplementation with 20 g/day of BC. To sum up, previous studies showed inconclusive results, which may be explained by the differences in methodological assumptions.

Current analysis also suggests no modulatory effect of BC on both pre- and post-exercise SIgA concentrations, even if changes in SIgA concentrations from baseline were incorporated into the pooled analysis. Taken together, these results suggest that a period of supplementation longer than 4 weeks and/or higher doses of BC split throughout the day may be necessary to induce significant changes in salivary IgA production. Moreover, it may be possible that the effect of BC may not be universal across athletes in different sports and in different age groups.

### 4.4. BC Safety

Based on the current literature, BC supplementation is considered to be well tolerated and safe for the human population. Reported adverse effects include only mild complaints, like nausea, diarrhea, flatulence, unpleasant taste, abdominal discomfort, which may disappear with time. Unfortunately there is no existing data for long term use of BC, so no conclusions can be made [9,10].

### 4.5. Strengths, Limitations, and Future Lines of Research

The strength of our work is the comprehensive search of published and unpublished studies, which included multiple electronic databases, scanning of bibliographies, and contact with authors to yield ten published studies. To date, there are only a limited number of randomised controlled trials in this area with small sample sizes. Another strength of this review is that it presents the first meta-analysis synthesising the effects of BC supplementation on the immunity of athletes and the physically active population.

This meta-analysis has several limitations that should be noted. The main limitation of this review is the scarcity of studies carried out in relation to BC supplementation in trained and physically active people (*n* = 10), which forced us to carry out the analyses by mixing data of both sexes, different training specificity and/or physical activity levels, different sample times, and different research protocols. Furthermore, the dose of supplied BC in the analysed studies and the duration of the supplementation were different and could influence the full interpretation of the collected data. Moreover, none of the included studies reported an a priori sample size calculation to detect the effect of BC on immunity, and it is likely that some experiments were unpowered. We are aware of the fact that there are more clinically useful biomarkers to assess in the field of nutritional immunology, but unfortunately there are not enough studies researching the effect of BC on these markers in physically active people to include them in this meta-analysis. Furthermore, it is important to underline that biomarkers should not be evaluated and/or interpreted in isolation.

Although the overall quality of the studies was sufficient, some trials included in this review were at some risk of bias and should be treated with caution. We do not consider these limitations to be sufficient to dismiss the findings of this review. However, future trials are urged to improve on the current reporting of randomised placebo-controlled trials on BC by adhering to Consolidated Standards of Reporting Trials (CONSORT) guidelines that facilitate critical appraisal and interpretation.

Future research projects should focus on the application of more clinically relevant immunological markers as well as determining the optimum dose and duration of supplementation in BC studies in physically active individuals. The next step is finding other possible mechanisms underlying the impact of BC on immunity in athletes. Moreover, it is important to conduct well-controlled randomised trials on a group of professional athletes.

## 5. Conclusions

This systematic review and meta-analysis shows that oral supplementation with BC has no significant effect on selected immunological outcomes including lymphocytes, neutrophils, serum IgG, serum IgA, and saliva SIgA in athletes and physically active people. This is highly important, especially in the presence of another meta-analysis confirming a positive effect of BC on URS in athletes. The present results suggest such effects may occur through other mechanisms, which remain unknown and should be studied using more specific and clinically relevant biomarkers.

## Figures and Tables

**Table 1:** Characteristics of included studies.

Study	Country	Study Design	Sample Size	Sex, Age (Years)	Inclusion Criteria	Exclusion Criteria	Duration	Intervention Dose	Intervention Additional Info	Placebo (PLA)
Mero et al. 1997 [17]	Finland	DB-RCT ^1^ and crossover	9	Only males 25 ± 2.5 ^2^	Athletes (sprinters, jumpers), drug-free	Use of supplements of amino acids, vitamins, minerals, or creatine monohydrate or any other sport supplement during the study phase	8 days	25/125 mL	Drink (containing BC) of 125 mL consumed in a splitdose twice per day (62.5 mL in the morning and 62.5 mL in the evening), not taken in the morning of the test training session but post-session. I treatment: 125 mL Bioenervi drink, II treatment: 25 mL Bioenervi (the drink of 125 mL containing the 25-mL Bioenervi supplement mixed with 100-mL placebo)	125 mL of ‘normal milk whey’ ^5^ per day
Mero et al. 2002 [11]	Finland	DB-RCT ^1^	30	BC: males 21.5 ± 0.7 ^3^, females 22.6 ± 1.6 ^3^; PLA: males 21.7 ± 1.9 ^3^, females 22.9 ± 2.6 ^3^	Athletes (track and field athletes, cross-country skiers, and orienteers), drug-free	Use of supplements of amino acids, vitamins, minerals, or creatine monohydrate, or any other supplement during the study phase	2 weeks	20 g	20 g of BC in a split dose four times per day (5 g)	20 g of maltodextrin per day
Crooks et al. 2006 [12]	New Zealand	DB-RCT ^1^	35	BC: males 46 (35–57) ^4^, females 43 (30–53) ^4^; PLA: males 48 (36–56) ^4^, females, 51 (41–58) ^4^	Recreational distance runners, pack runs ≥ 1 week, marathon training over last 5 years, age < 60 year	Lactose intolerance, allergy to cows milk, use of whey-protein supplements, treatment for any diagnosed condition	12 weeks	10 g	26 g of powdered sachets/day corresponding to 10 g of BC (chocolate powder with 125 ml water)	Skim milk
Shing et al. 2007 [18]	Australia	DB-RCT ^1^	29	Only males BC: 29 ± 1 ^3^ PLA: 27 ± 2 ^3^	Athletes (cyclists), racing competitively ≥ 2 seasons, consistent training volumes ≥ 2 months	Use of dietary supplements for 1 month prior to study	5 weeks	10 g	10 g of BC per day in the morning with 50 mL water + 100 mL milk.	10g of whey protein per day
Davison et al. 2010 [14]	United Kingdom	DB-RCT ^1^	20	Only males 25.0 ± 5 ^2^	None reported	None reported	4 weeks	20 g	20 g of BC per day	PLA containing an isoenergetic and isomacronutrient mixture of milk protein concentrate
Crooks et al. 2010 [13]	New Zealand	DB-RCT ^1^	25	BC: males 17 ± 1 ^3^, females 20 ± 1 ^3^; PLA: males 19 ± 1 ^3^, females 18 ± 1 ^3^	Athletes (swimmers), participating in training program prior to The Auckland Swimming Championships	Lactose intolerance, allergy to cows’ milk, use of whey-protein, immunological-modulating supplements, treatment for any diagnosed condition	10 weeks	20 g	52 g of powdered sachets per day corresponding to 20 g of BC per day, in a split dose twice per day: 10 g morning & evening, with 125 mL water	Skim milk powder
Carol et al. 2011 [24]	The Netherlands	DB-RCT ^1^ and crossover	9	Only males 27.3 ± 4.5 ^2^	Athletes (cyclists), >2 years of cycling experience, training >3 times/week during > 9 months/year, refraining from using dietary supplements	Allergy to cow’s milk or a known immune disease, signs of infection during the month preceding the study, treatment for any medical condition, use of any drugs, or consume more than two alcoholic beverages per day	10 days	25 g	12.5 g of BC twice a day, with a glass of cold milk or cold buttermilk	Skim milk powder
Shing et al. 2013 [19]	Australia	DB-RCT ^1^	10	Only males BC: 22 ± 3 PLA: 23 ± 2 ^3^	Athletes (cyclists), racing competitively ≥3 seasons, consistent training volumes ≥ 2 months	None reported	8 weeks and 5 days	10 g	10 g of BC per day, morning with 50 mL water + 100 mL milk	10 g Whey protein concentrate per day
Jones et al. 2014 [15]	United Kingdom	DB-RCT ^1^	53	Only males BC: 31 ± 14 ^2^ PLA: 32 ± 13 ^2^	Recreationally active people, ≥3 h moderate-vigorous endurance exercise/ week	Smoking, use of medication or other supplements, infectious illness in 4 weeks prior to study	12 weeks	20 g	20 g of BC per day, in a split dose: 10 g with morning & evening meal	Isoenergetic/isomacronutrient
Jones et al. 2015 [16]	United Kingdom	DB-RCT ^1^	20	Only males 28 ± 8 ^2^	Recreationally active men	Smoking, allergy to dairy products and reported symptoms of infection or use of any medication or dietary supplements 4 weeks prior to commencement of the study	4 weeks	20 g	20 g of BC per day, in a split dose: 10 g morning & evening on an empty stomach	Isoenergetic/isomacronutrient

^1^ DB-RCT randomized double-blind placebo-controlled trial; ^2^ Mean ± SD, ^3^ Mean ± SE, ^4^ Median (range), ^5^ The phrase used by the original authors.

## Supplementary Materials

The following are available online at https://www.mdpi.com/2072-6643/12/4/1023/s1, Figure S1. Funnel plot of the random-effects meta-analysis of changes in lymphocytes concentration; Figure S2. Funnel plot of the random-effects meta-analysis of changes in neutrophils concentration; Figure S3. Funnel plot of the random-effects meta-analysis of changes in IgA concentration; Figure S4. Funnel plot of the random-effects meta-analysis of changes in IgG concentration; Figure S5. Funnel plot of the random-effects meta-analysis of changes in pre-exercise SIgA concentration; Figure S6. Funnel plot of the random-effects meta-analysis of changes in post-exercise SIgA concentration; Figure S7. Funnel plot of the random-effects meta-analysis of changes from baseline to post-supplementation in pre-exercise SIgA concentration; Figure S8. Funnel plot of the random-effects meta-analysis of changes from baseline to post-supplementation in post-exercise SIgA concentration.
