# Supplementary material for: Immunological Outcomes of Bovine Colostrum Supplementation in Trained and Physically Active People: A Systematic Review and Meta-Analysis
Source: Nutrients. 2020 Apr 8;12(4):1023. doi: 10.3390/nu12041023 (PMC7231218; doi:10.3390/nu12041023)
Supplement: Supplementary file 1 [file nutrients-12-01023-s001.pdf]

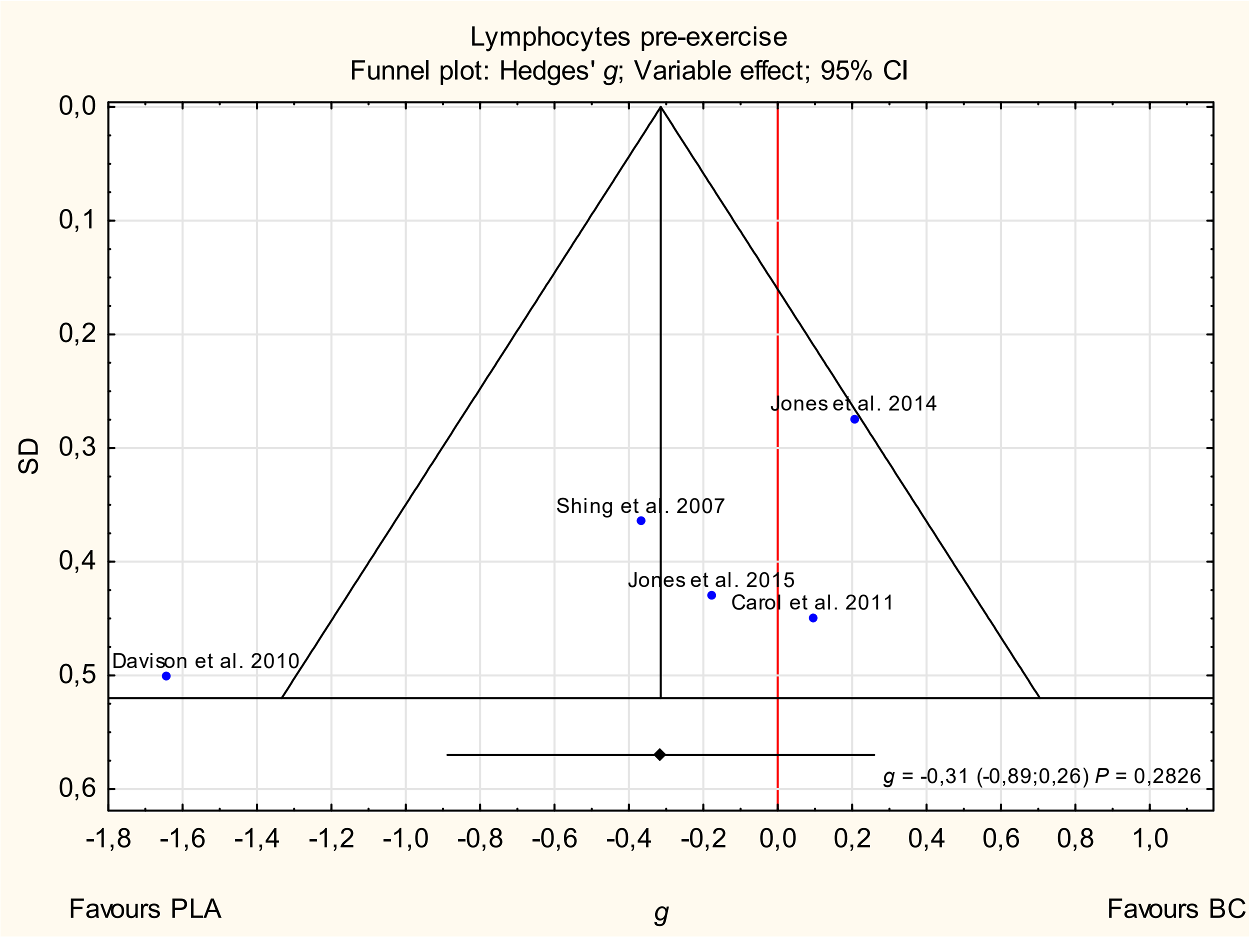

Figure S1. Funnel plot of the random-effects meta-analysis of changes in lymphocytes concentration. Abbreviations: BC – bovine colostrum, PLA – placebo.

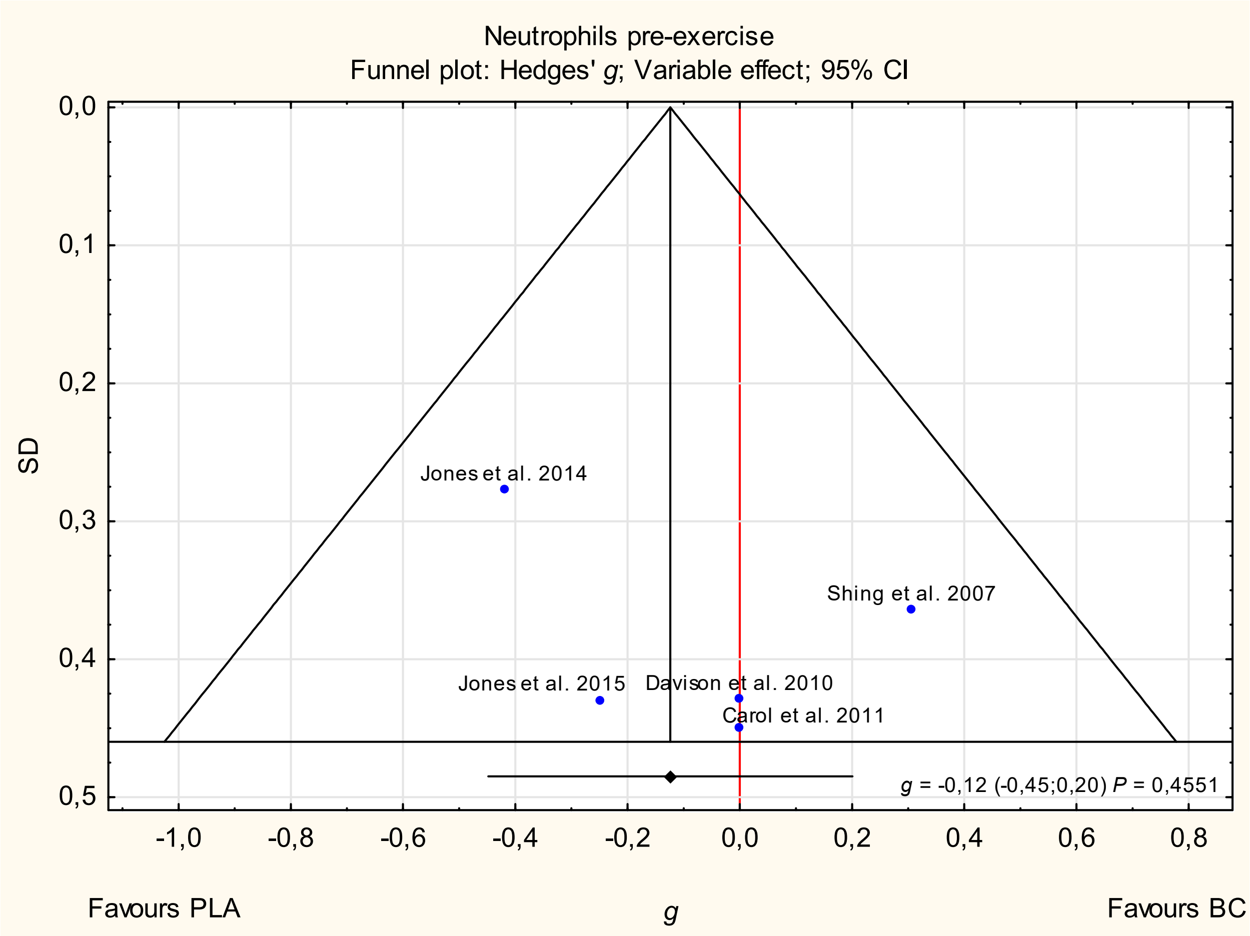

Figure S2. Funnel plot of the random-effects meta-analysis of changes in neutrophils concentration. Abbreviations: BC – bovine colostrum, PLA – placebo.

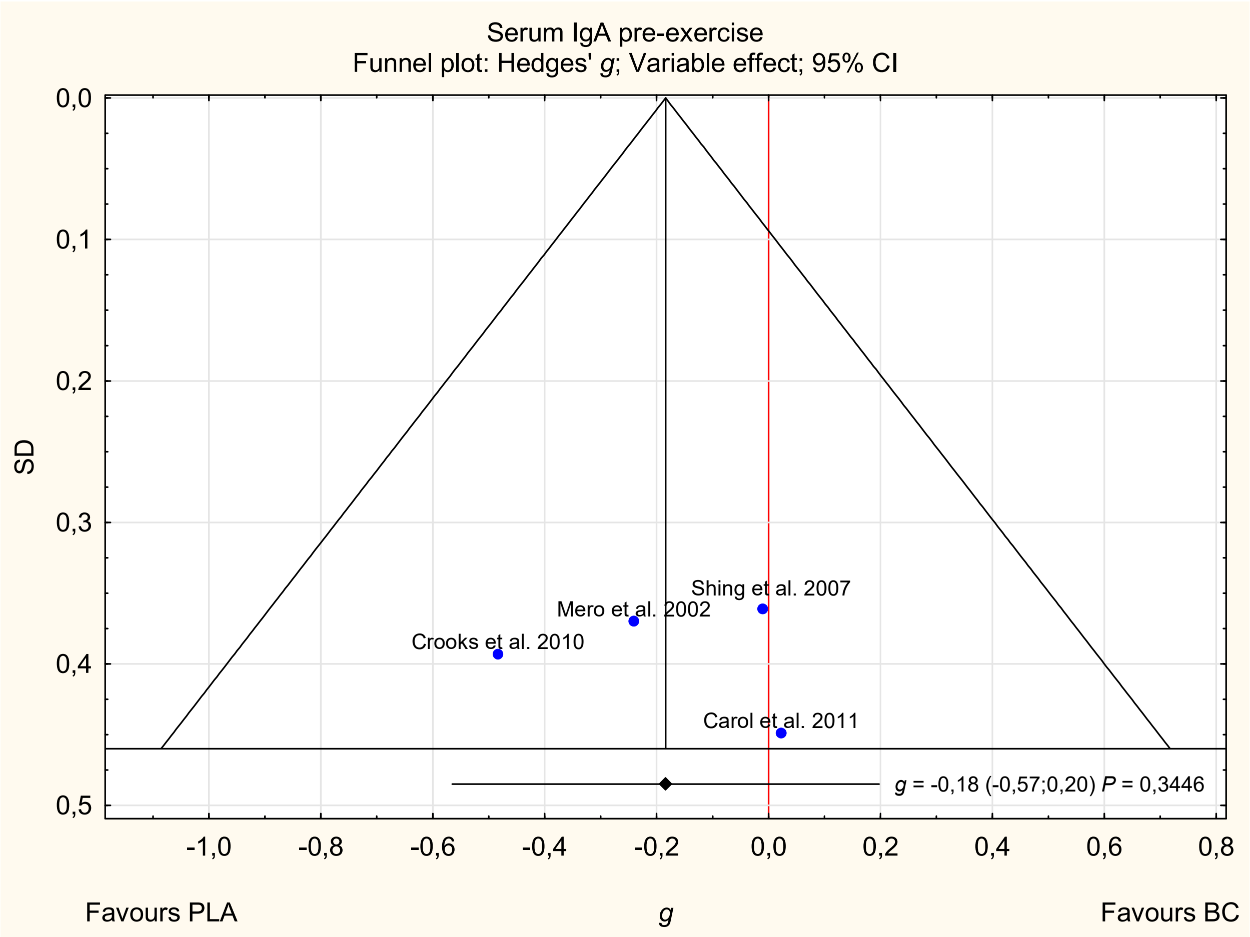

Figure S3. Funnel plot of the random-effects meta-analysis of changes in IgA concentration. Abbreviations: BC – bovine colostrum, IgA – Immunoglobulin A, PLA – placebo.

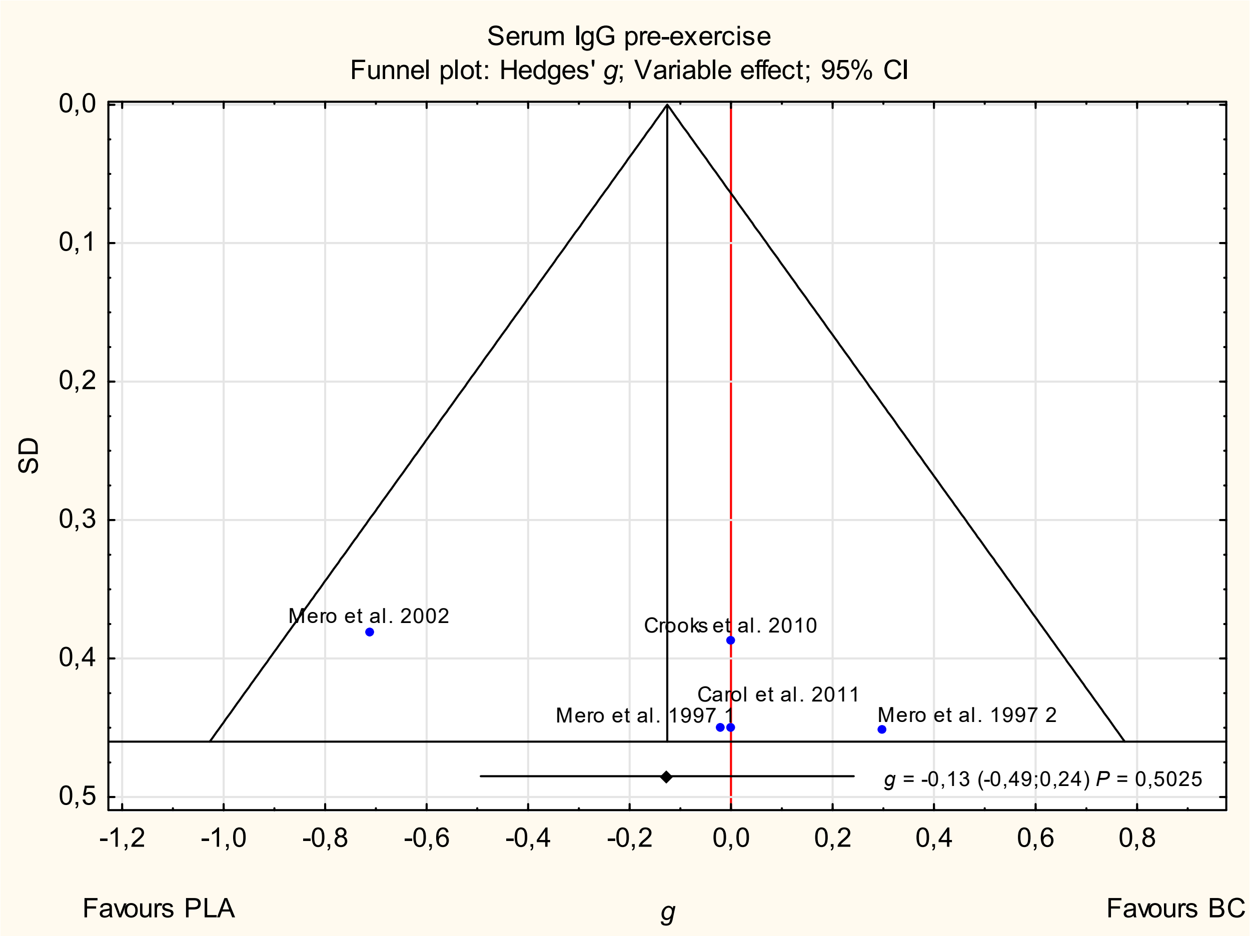

Figure S4. Funnel plot of the random-effects meta-analysis of changes in IgG concentration. 1 – the first dose from Mero et al. 1997 study, 2 – the second dose from Mero et al. 1997 study. Abbreviations: BC – bovine colostrum, IgG – Immunoglobulin G, PLA – placebo.

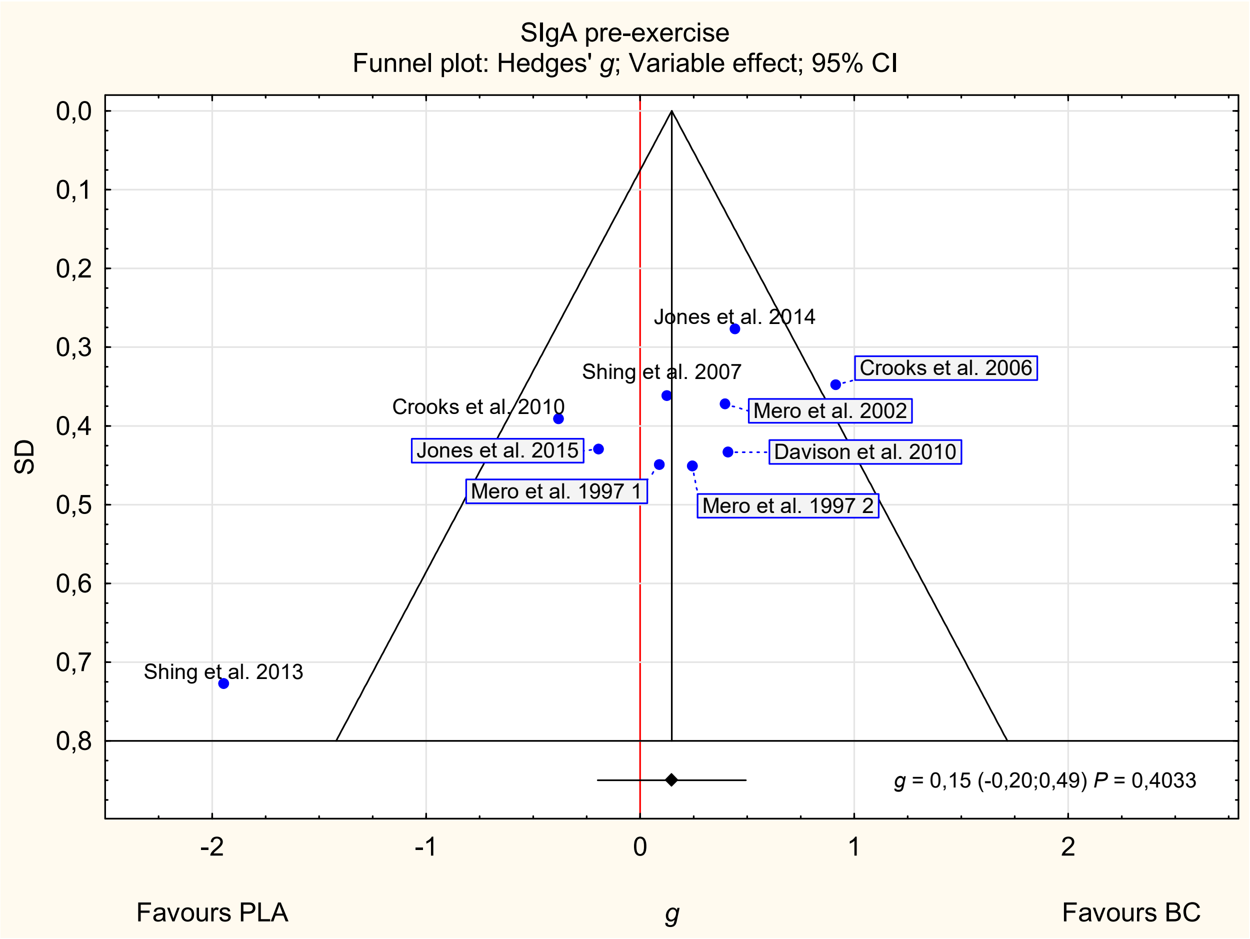

Figure S5. Funnel plot of the random-effects meta-analysis of changes in pre-exercise SIgA concentration. 1 – the first dose from Mero et al. 1997 study, 2 – the second dose from Mero et al. 1997 study. Abbreviations: BC – bovine colostrum, SIgA – secretory Immunoglobulin A, PLA – placebo.

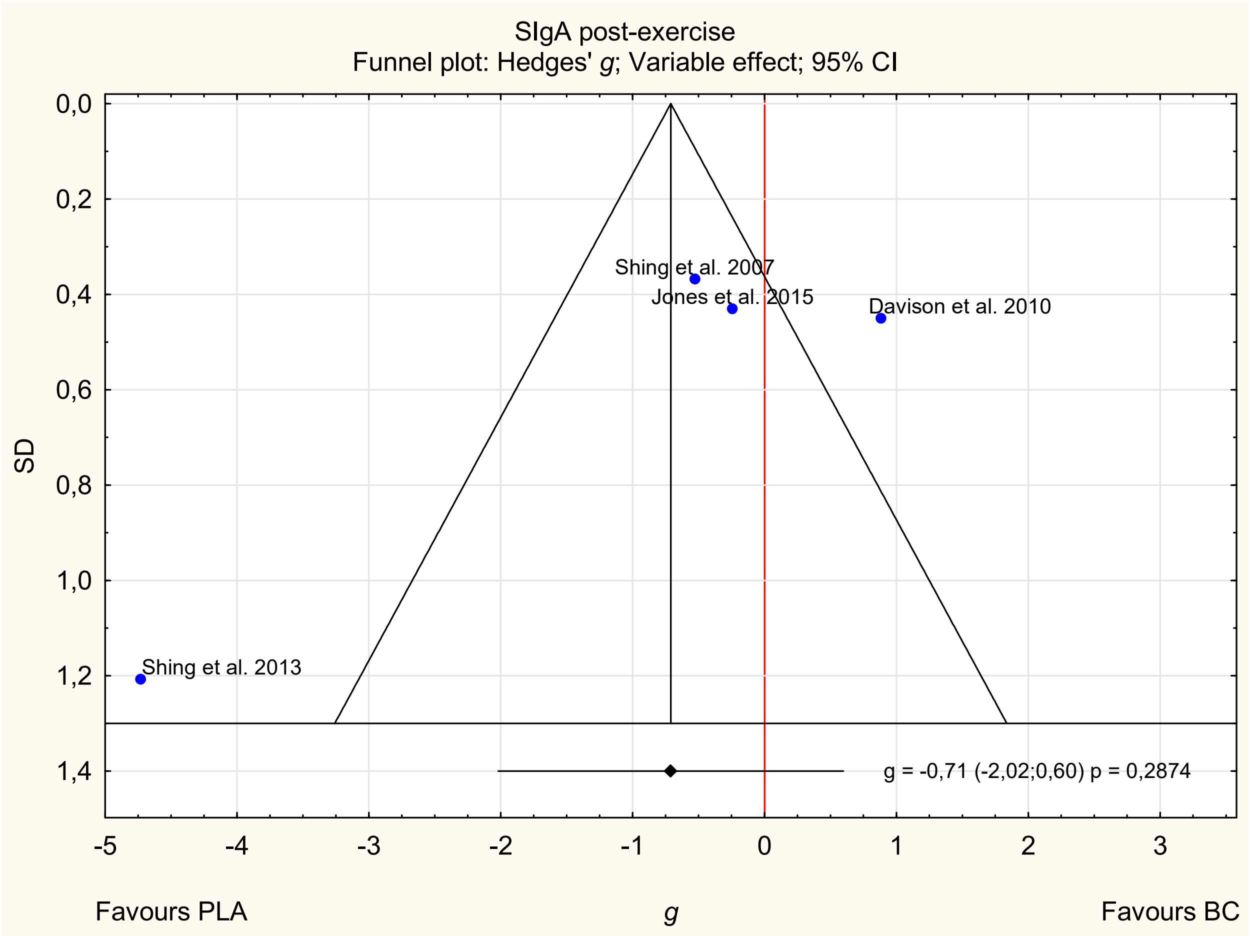

Figure S6. Funnel plot of the random-effects meta-analysis of changes in post-exercise SIgA concentration. Abbreviations: BC – bovine colostrum, SIgA – secretory Immunoglobulin A, PLA – placebo.

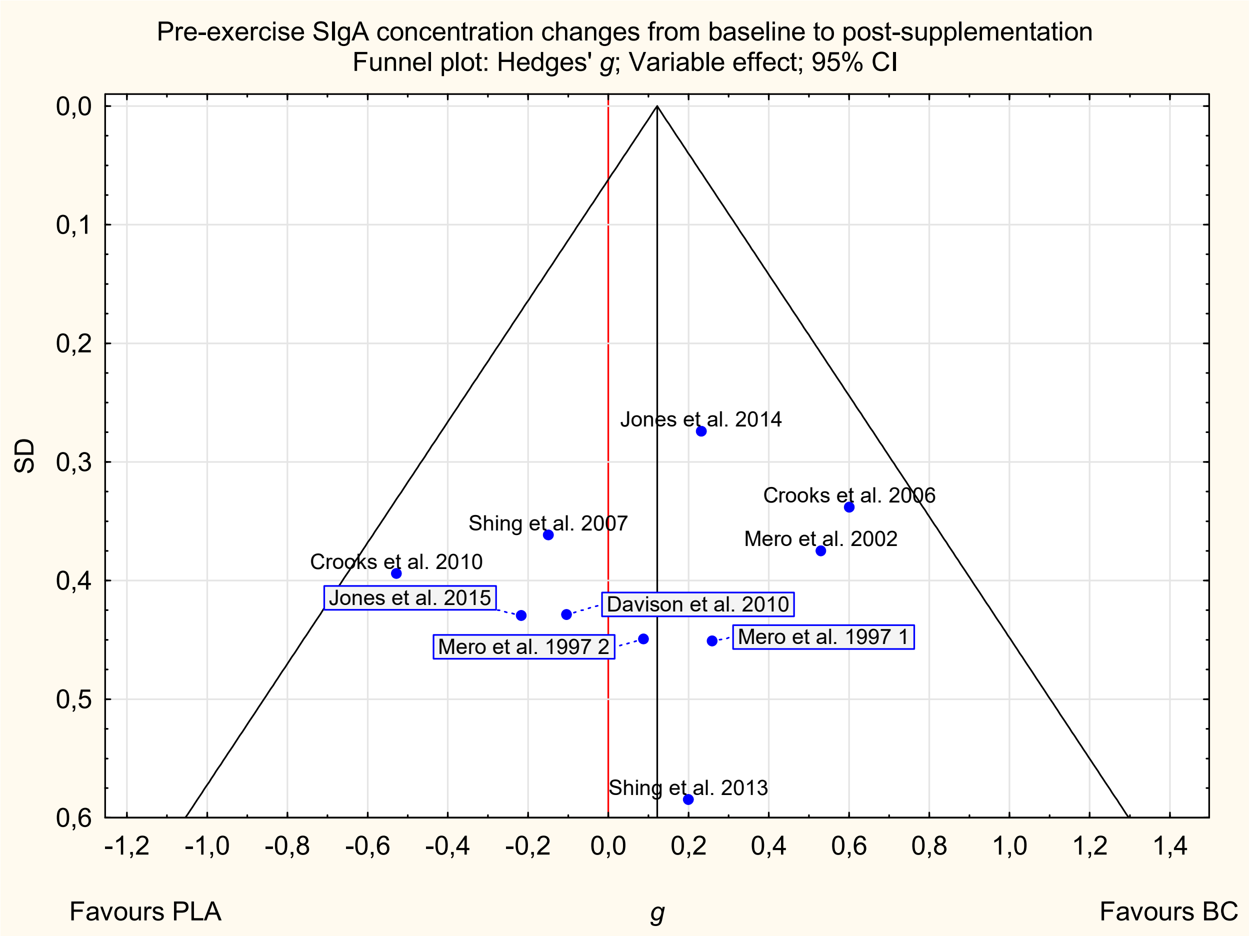

Figure S7. Funnel plot of the random-effects meta-analysis of changes from baseline to post-supplementation in pre-exercise SIgA concentration.

1 – the first dose from Mero et al. 1997 study, 2 – the second dose from Mero et al. 1997 study. Abbreviations: BC – bovine colostrum, SIgA – secretory Immunoglobulin A, PLA – placebo.

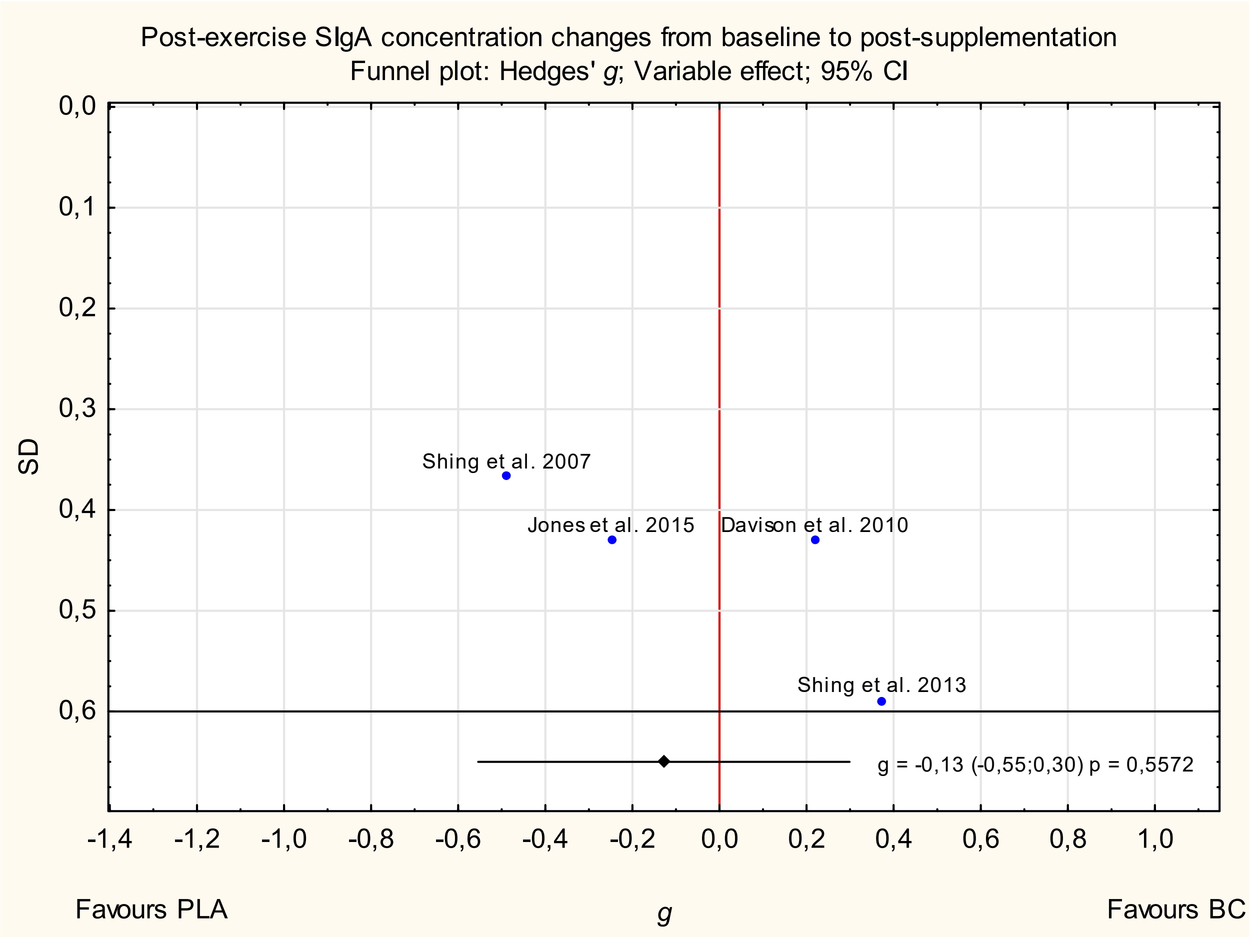

Figure S8. Funnel plot of the random-effects meta-analysis of changes from baseline to post-supplementation in post-exercise SIgA concentration.

Abbreviations: BC – bovine colostrum, SIgA – secretory Immunoglobulin A, PLA – placebo.
